# Comment on ‘elevated C-reactive protein to albumin ratio is a promising predictive biomarker for prognosis in patients with renal cell carcinoma’

**DOI:** 10.1080/07853890.2026.2640286

**Published:** 2026-03-06

**Authors:** Jianfeng Shen

**Affiliations:** Department of Pharmacy, Shaoxing Central Hospital, Shaoxing University, Shaoxing, China

Dear Editor

I read with great interest the recent systematic review and meta-analysis by Xu et al. [1] investigating the prognostic value of the C-reactive protein-to-albumin ratio (CAR) in patients with renal cell carcinoma (RCC). The authors are commended for their efforts in synthesizing evidence on inflammation-related biomarkers in oncology. However, upon careful examination of the methodological details, I identified several potential methodological concerns and conceptual inconsistencies that may substantially affect the robustness and clinical applicability of their conclusions.

First, I was puzzled by the authors’ statement that Hardy–Weinberg equilibrium (HWE) testing was performed in the control groups of the included studies. As is well established, HWE is a principle in population genetics used to assess the stability of allele frequencies and is applicable only to genetic polymorphism studies. In contrast, CAR is a biochemical ratio derived from serum C-reactive protein (CRP) and albumin—both of which are dynamically influenced by inflammatory, metabolic, and environmental factors—and is not a genetic marker. Applying HWE testing to such a non-genetic, continuous biomarker lacks biological plausibility and may reflect an inappropriate application of methodology. Clarification regarding the rationale for this analytical step would be helpful.

Second, Table 2 reports CAR cutoff values with units labeled as ‘ng/mL’, ranging from 0.025 to 0.11 ng/mL. This raises a significant concern regarding unit consistency. In routine clinical practice, CRP is typically measured in mg/L (or μg/mL), while albumin is reported in g/L; consequently, CAR is a dimensionless ratio. Reported CAR values in the literature generally fall within the range of 0.03 to 2.0. A CRP concentration of 0.025 ng/mL—as implied by the stated units—would be several orders of magnitude below the detection limit of even high-sensitivity assays (which operate in the μg/L or mg/L range). This apparent discrepancy in units or numerical scale may stem from a unit labeling error, scaling issue (e.g. mg/L vs. g/L), or inconsistency in data extraction and reporting across the included studies. Verification of the accuracy of data extraction and unit standardization would enhance the interpretability of the findings.

Third, the manuscript states that all included studies achieved a ‘Cochrane score of 10 or higher’. This phrasing is misleading. The Cochrane Risk of Bias (RoB) tool does not employ a numerical scoring system—particularly not a 10-point scale—and its standard versions (RoB 1.0 and RoB 2.0) are specifically designed for randomized controlled trials (RCTs) [2]. As shown in Table 1, all 10 studies included in this meta-analysis are retrospective observational studies. Therefore, the use of the term ‘Cochrane score’—and its quantification as a numerical value—appears inconsistent with standard methodological practice. For observational studies, validated instruments such as the Newcastle–Ottawa Scale (NOS) would be more methodologically suitable for quality assessment. It is possible that the NOS was applied, but the terminology used may have caused confusion.

Finally, I note that the authors employed diagnostic test accuracy (DTA) metrics—including sensitivity, specificity, and summary receiver operating characteristic (SROC) curves—to evaluate time-to-event outcomes such as overall survival. While this approach has occasionally appeared in the literature, it conflates fundamentally distinct statistical frameworks: prognostic modeling (which estimates risk over time) versus diagnostic modeling (which assesses binary classification at a single timepoint). Hazard ratios (HRs) are the standard metric in prognosis meta-analyses as they directly quantify the effect of a biomarker on survival over time. In contrast, diagnostic accuracy methods may be misleading unless a clear timepoint-based dichotomization method is specified. A synthesis based on hazard ratios (HRs) with 95% confidence intervals would better align with prognostic research standards and strengthen the validity of the conclusions.

In summary, while I appreciate the authors’ initiative in exploring CAR as a potential prognostic biomarker in RCC, the aforementioned methodological issues warrant clarification and, where appropriate, correction. I respectfully encourage the authors to reconsider their analytical approach and hope to see a revised analysis grounded in methodological principles more congruent with prognostic research paradigms.

## Data Availability

There is no data associated with this research.
